# Relating family satisfaction to the care provided in intensive care units: quality outcomes in Saudi accredited hospitals

**DOI:** 10.5935/0103-507X.20170018

**Published:** 2017

**Authors:** Mohamed Saad Mahrous

**Affiliations:** 1 College of Dentistry, Taibah University - Madinah, Arábia Saudita.

**Keywords:** Professional family relations, satisfaction, Critical care, Quality of health care, Saudi Arabia

## Abstract

**Objectives:**

This study aims to identify the satisfaction levels of the family members of patients in intensive care units.

**Methods:**

This is a cross-sectional analytical study. General intensive care units offer a variety of services to clinical and surgical patients. For the purpose of this study, a trained interviewer communicated with the families of patients, either before or after visiting hours.

**Results:**

The study included 208 participants: 119 (57.2%) males and 89 (42.8%) females. Seventy-three (35.1%) of the patients attended a private hospital, and 135 (64.9%) attended a public hospital in the city of Al Madinah Al- Munawarah. All of the participants were either family members or friends of patients admitted to the intensive care units at the hospitals. The responses of both groups yielded low scores on the satisfaction index. However, a relatively high score was noted in response to questions 2, 6, and 10, which concerned the care that was extended by the hospital staff to their patients, the courteous attitude of intensive care unit staff members towards patients, and patients' satisfaction with the medical care provided, respectively. A very low score was obtained for item 11, which was related to the possibility for improvements to the medical care that the patients received. Overall, greater satisfaction with the services offered by the public intensive care units was reported compared to the satisfaction with the services offered by the private intensive care units.

**Conclusion:**

An overall low score on the satisfaction index was obtained, and further studies are recommended to assess the current situation and improve the satisfaction and quality of care provided by intensive care units.

## Abstract (pt)

**Objetivo:**

Identificar os níveis de satisfação dos familiares de pacientes internados em unidades de terapia intensiva.

**Métodos:**

Este foi um estudo analítico transversal. As unidades gerais de terapia intensiva oferecem uma variedade de serviços a pacientes clínicos e cirúrgicos. Para as finalidades deste estudo, um entrevistador treinado fez contato com familiares dos pacientes, antes ou após o horário de visita.

**Resultados:**

O estudo incluiu 208 participantes, sendo 119 (57,2%) do sexo masculino. Dentre os pacientes, 73 (35,1%) se encontravam em um hospital particular e 135 (64,9%) em um hospital público na cidade de Al Madinah Al-Munawarah. Todos os participantes eram membros da família ou amigos de pacientes admitidos às unidades de terapia intensiva dos hospitais. As respostas de ambos os grupos proporcionaram escores baixos nos índices de satisfação. Observou-se, porém, um escore relativamente elevado nas respostas às questões 2, 6 e 10, concernentes, respectivamente, aos cuidados proporcionados pela equipe do hospital a seus pacientes, à atitude de cortesia dos membros da equipe das unidades de terapia intensiva para com os pacientes, e à satisfação dos pacientes com os cuidados médicos proporcionados. O escore foi muito baixo para o item 11, relacionado à possibilidade de melhorias nos cuidados médicos que os pacientes recebiam. Em geral foi relatada uma satisfação maior com os serviços oferecidos pelas unidades de terapia intensiva dos hospitais públicos, em comparação com a satisfação com os cuidados oferecidos pelas unidades de terapia intensiva dos hospitais privados.

**Conclusão:**

Em geral, foi obtido um escore baixo nos índices de satisfação, recomendando-se a realização de outros estudos para avaliar a situação atual, e melhorar a satisfação e a qualidade dos serviços fornecidos pelas unidades de terapia intensiva.

## INTRODUCTION

Patient safety is the cornerstone of a successful health system and policy, and measuring the quality of care and associated outcomes is one method that is performed to establish patient safety. When assessing health services, health care organizations consider patient satisfaction to be a quality indicator. Although some studies suggest that patient satisfaction is not associated with other quality indicators, which potentially raises questions about its importance,^(1)^ increased satisfaction among patients has been associated with a higher clinical performance when conducting quality measures to assess certain diagnoses.^(2)^

In intensive care units (ICUs), the type and intensity of the disease, in addition to the patient's level of consciousness, can make it difficult to determine the patient's level of satisfaction by consulting them directly. Thus, family members serve as a valuable source of information in this context.^(3)^ Harvey^(4)^ confirms that in a critical care situation, the treatment of the patient has a direct impact on family satisfaction, which can thus be used as an alternative measure of patient satisfaction in ICU settings.^(5)^

When patients are admitted to the ICU due to an acute medical condition, their family members experience psychological stress, emotional disturbance, shock, depression and anxiety,^(3,6)^ even if the patient is being admitted to a highly ranked hospital. Undoubtedly, despite the challenges they face, family members are particularly essential providers of support for patients and play an important role in their recovery. According to Neves et al., assessing the level of satisfaction of the family members of ICU patients is a crucial aspect for health professionals to consider when providing care to reduce the pain and suffering of critically ill patients.^(7)^ Thus, we should consider their role beyond that of merely being visitors to the ICU.^(3,8)^

Evidence in the literature reveals the extent and importance of family members' satisfaction with patient care.^(4,7,9)^ Previous studies have indicated that fulfilling the needs of patients' families results in better quality of care for the patient, increasing satisfaction.^(10,11)^ Notably, multiple studies describe family members' satisfaction with care when their relatives are receiving care in the ICU.^(10,11)^ A study in Australia revealed that fulfilling the needs of patients' family members makes them more willing to participate in the care of the patient following discharge.^(10)^

In Saudi Arabia, there are no published data regarding the level of satisfaction among patients or their family members in relation to ICUs. In addition to filling this gap, another advantage of conducting such a study in Saudi Arabia is that it would make it possible to compare the satisfaction level of the services provided by ICUs of government run hospitals to that provided by ICUs of private hospitals.

The aim of this study is to identify the degree of satisfaction among the family members of patients who are admitted to ICUs, concentrating on their communication with the health care teams, at both government and privately owned hospitals. The degree of satisfaction among family members is one of the quality indicators used to measure service levels at ICUs in the city of Al-Madinah Al-Munawarah.

## METHODS

This is a cross-sectional analytical study. The study was implemented in the general ICUs at two hospitals (one is governmentally run, and the other is privately run) that are located in Al-Madinah Al-Munawarah, Saudi Arabia. The ICUs provide services for clinical and surgical patients. A trained interviewer conducted interviews with the family members of patients around the visiting hours. All of the study participants were over 18 years old and had a relative who had been admitted to the ICU for at least 24 hours. Only one family member was interviewed for each patient.

All of the study participants received information concerning the aim of the study and the voluntary nature of their decision to participate in it. Those relatives who agreed to voluntarily participate in the study were given a self-administered questionnaire to complete and return to the interviewer. The researcher met the participants in the ICU waiting areas at a time when visits were permitted. The interviewer offered assistance to those who could not read or write by filling in their answers for them.

The Taibah University College of Dentistry Research Ethics Committee (TUCDREC) revised and approved the study (Approval number: TUCDREC/20160110), including the waiver documentation pertaining to informed consent. This was granted on the basis that the study would rely on a self-administered questionnaire. After reading the first page, which introduced the study aim and stated the voluntary nature of their participation, only those who agreed to participate in the study completed the questionnaire.

This study used an Arabic translated version of a questionnaire, which was adapted from a modified, validated instrument that was devised by Johnson et al.^(12)^ based on the Critical Care Family Needs Inventory (CCFNI) originally described by Molter.^(13)^ A back translation into English was prepared to ensure that the Arabic translation matched the English version. A technique that is similar to this was previously explained by Fumis.^(14)^ The questionnaire included 14 questions, with replies arranged into four responses: 1 (almost all the time), 2 (most of the time), 3 (only part of the time), and 4 (never). Each question was scored as one if the reply was 1 or 2 (indicating satisfaction) or zero if the reply was 3 or 4 (indicating dissatisfaction). An exception was made for questions 11 and 14, in which the scoring system was inverted. The satisfaction level was calculated by adding together the scores for all of the questions, with the minimum value being zero (extreme dissatisfaction) and the maximum value being fourteen (extreme satisfaction).^(3)^

The Statistical Package for Social Science (SPSS) software version 23 was used for the statistical analysis of the data. Descriptive statistics were prepared using measures of central tendency and dispersion. In addition, qualitative data were prepared, and the results were reported as the frequencies and percentages. However, variables that affected distribution (age of the patients and relatives, length of stay in the ICU until the interview, and number of visits) were ranked at two levels and divided into groups according to the median. The level of family members' satisfaction is expressed as the median and interquartile interval (P25 - P75). The Mann-Whitney test was used to correlate the family members' level of satisfaction with other variables, and the significance level was set at the 0.05 level.

## RESULTS

This study was conducted over a period of three months, from February 2016 to April 2016. In total, 208 family members participated: 73 (35.1%) from a private hospital and 135 (64.9%) from a government hospital in the city of Al-Madinah Al-Munawarah. Therefore, family members and friends of patients who were admitted to ICUs of both private and government hospitals were represented.

Table 1 shows the sociodemographic characteristics of the study groups that were interviewed from the private and government hospitals.

**Table 1:** Sociodemographic characteristics of the study group

Variable	(N = 208) N (%)
Health care facility	
Private	73 (35.1)
Governmental	135 (64.9)
Sex	
Male	119 (57.2)
Female	89 (42.8)
Cause of admission	
Medical	100 (48.1)
Surgical	108 (51.9)
Level of kinship	
Father/mother	69 (33.2)
Spouse	8 (3.8)
Son/daughter	24 (11.5)
Brothers/sisters	67 (32.2)
Others	40 (19.2)
Education level	
Incomplete high school	68 (32.7)
Complete high school	48 (23.1)
Incomplete college	20 (9.6)
Complete college	72 (34.6)
Age	
Mean ± SD	37 ± 20.59
Median	31
Percentile 25	24
Percentile 50	31
Percentile 75	46
ICU length of stay	
Mean ± SD	21.79 ± 21.325
Median	17
Percentile 25	6
Percentile 50	17
Percentile 75	30

SD - standard deviation; ICU - intensive care unit.

Table 2 shows that there was a low satisfaction index score among both groups; however, a relatively high satisfaction index score is noted in response to questions 2, 6, and 10, which concerned statements regarding the feeling of care provided by the hospital staff to the patients, the courteousness of ICU staff members, and their satisfaction with the medical care that the patient received. Thus, a very low satisfaction index was noted for question 11, which inquired about the need to improve aspects of the medical care that the patient received.

**Table 2:** Satisfaction index of family members for the questions of the Critical Care Family Needs Inventory

Questions	Almost all the time	Most of the time	Only part of the time	None of the time	Satisfaction index*
1. Do you feel that the best possible care is being given to the patient?	36.1	21.6	25	17.3	57.7
2. Do you feel that the hospital personnel care about the patient?	44.7	29.3	24	1.9	74
3. Have the explanations given to you about the patient’s clinical condition been in terms you can understand?	26.4	25	23.6	25	51.4
4. Do you feel that you have been given honest information about the patient’s condition?	28.4	26.9	31.3	13.5	55.3
5. Do you understand what is happening to the patient and why things are being done?	24.5	25	32.7	17.8	49.5
6. Have the intensive care unit staff members been courteous to you?	41.8	33.2	21.2	3.8	75
7. Have any of the staff members shown interested in how you are doing?	22.6	13.5	11.5	52.4	36.1
8. Do you believe that someone will call you at home with any major or significant change in the patient’s condition?	32.2	15.4	13.5	38.9	47.6
9. Have the hospital personnel explained the equipment being used?	33.7	12	17.8	36.5	45.7
10. I am very satisfied with the medical care the patient receives.	38	25.5	17.3	19.2	63.5
11. There are some things about the medical care the patient receives that could be better.	41.8	37	13.5	7.7	21.2
12. Do you feel comfortable visiting the patient in the intensive care unit?	24.5	27.4	36.5	11.5	51.9
13. Is the waiting room comfortable?	28.4	23.6	26.9	21.2	51.9
14. Do you feel alone and isolated in the waiting room?	30.3	13.5	32.7	23.6	56.3

* The satisfaction index was calculated based on the sum of replies received at levels 1 and 2, except for questions 11 and 14, where it was calculated based on the sum of replies reached at levels 3 and 4. The results are expressed as the %.

Table 3 shows a comparison that was made between the satisfaction indices attained for the private hospital's ICU services and the government hospital's ICU services. Either no statistically significant difference was noticed, or greater satisfaction was professed concerning the services offered by the government ICUs, as apparent from questions 1, 2, 4, 5, 6, 7, 9, 10, 11, and 14.

**Table 3:** Comparison of the satisfaction of family members between the intensive care units of private and governmental hospitals

Questions	Private N = 73 N (%)	Governmental N = 135 N (%)	p value
1. Do you feel that the best possible care is being given to the patient?	25 (34.2)	95 (70.4)	< 0.001*
2. Do you feel that the hospital personnel care about the patient?	45 (61.6)	109 (80.7)	0.003*
3. Have the explanations given to you about the patient’s clinical condition been in terms you can understand?	36 (49.3)	71 (52.6)	0.380 (NS)
4. Do you feel that you have been given honest information about the patient’s condition?	32 (43.8)	83 (61.5)	0.011*
5. Do you understand what is happening to the patient and why things are being done?	20 (27.4)	83 (61.5)	< 0.001*
6. Have the intensive care unit staff members been courteous to you?	41 (56.2)	115 (85.2)	< 0.001*
7. Have any of the staff members shown interested in how you are doing?	12 (16.4)	63 (46.7)	< 0.001*
8. Do you believe that someone will call you at home with any major or significant change in the patient’s condition?	36 (49.3)	63 (46.7)	0.413 (NS)
9. Have the hospital personnel explained the equipment being used?	16 (21.9)	79 (58.5)	< 0.001*
10. I am very satisfied with the medical care the patient receives.	29 (39.7)	103 (76.3)	< 0.001*
11. There are some things about the medical care the patient receives that could be better.	24 (32.9)	20 (14.8)	0.002*
12. Do you feel comfortable visiting the patient in the intensive care unit?	33 (45.2)	75 (55.6)	0.1 (NS)
13. Is the waiting room comfortable?	41 (56.2)	67 (49.6)	0.225 (NS)
14. Do you feel alone and isolated in the waiting room?	49 (67.1)	68 (50.4)	0.014*

NS - not significant. The satisfaction index was calculated based on the sum of replies received at levels 1 and 2, except for questions 11 and 14, where it was calculated based on the sum of replies reached at levels 3 and 4.

* Significant at a 0.05 level using Fisher’s Exact test.

The median level of satisfaction for the family members is 7 (4 - 11). A higher median satisfaction level was calculated for government ICUs and lower education levels, and this was statistically significant. However, no statistically significant difference was found when comparing gender, cause of admission and number of visits (p value > 0.05) (Table 4).

**Table 4:** Factors associated with the satisfaction of family members

Factor	Median (percentiles)	p value
Health care facility		0.001*
Private	5 (1 - 11)	
Governmental	8 (5 - 11)	
Gender		0.06 (NS)
Male	8 (5 - 11)	
Female	7 (4 - 11)	
Cause of admission		0.143 (NS)
Medical	7 (4 - 11)	
Surgical	7 (4 - 11)	
Number of visits		0.904 (NS)
Up to 4	6.5 (4 - 12.5)	
5 or more	7 (4 - 11)	
Education level		< 0.001*
Up to high school	8 (5 - 11)	
College of more	6 (2 - 10)	

NS - not significant.

* Mann-Whitney test, which was significant at a 0.01 level.

## DISCUSSION

The involvement of family members or relatives in assessing the satisfaction of ICUs provides important guidance to assist with assessing the communication practices in these units. Research that studies the factors of stress for patients admitted to the ICU and health professionals working in the ICU is needed. Recently, authors have noted the advantage of assessing the needs of the family members or companions of admitted patients and their level of satisfaction with the care provided by ICUs.^(2,6,13)^ Other studies have claimed that a higher family satisfaction with an ICU is associated with several domains of a better organizational/safety culture.^(15,16)^

A low satisfaction index among both groups indicates that more work is needed in the ICU units in the city of Al-Madinah Al Munawarah to increase satisfaction by improving the quality of care. However, the relatively high satisfaction index that was obtained for questions 2, 6, and 10, which concerned the level of care of the hospital staff toward patients, the courtesy of ICU staff members, and the satisfaction with the medical care that the patient received, is encouraging. This is also reflected in the very low score for item 11, which regarded the need for improvements to the medical care.

Other studies that emphasize the importance of the role of ICU health professionals in caring for the patient explain how services are perceived and their feelings when administering these services. These are directed mainly towards the patient, without consideration for the views of their relatives or family members.^(2,3,16)^

A high level of stress in the working conditions of the ICU is known to be encountered, and these working conditions contribute to the daily suffering and death among patients. The reaction to work-associated stress takes the form of Burnout Syndrome and is manifested as a remoteness among professionals and staff who are directly involved with care because indifference creates a feeling of safety.^(3)^ However, this could lead to a decline in the quality of care given to the patient and/or their families.^(17)^

The data shown in table 2 present the measures that we need to focus on to fulfill the needs of family members to increase their level of satisfaction with and understanding of the care given in an ICU setting. Some of the measures proposed include: (i) assigning a room to a social worker to explain the work policy in the ICU to families, (ii) arranging a private meeting with doctors to explain patients' conditions and treatment plans, (iii) offering educational videos about ICU routines that could be shown to family members in the waiting room prior to visits, (iv) providing leaflets or brochures, and (v) posting diagrammatic posters with the descriptions and details of the function and terminology associated with the equipment used in the unit.

The results show a lower level of satisfaction (Table 3) with the services offered by the private hospital for a majority of the questions. This can be attributed to the following: (i) such hospitals in Saudi Arabia often have fewer professional staff members working in the ICU, (ii) the number of patients who are admitted to such units and their medical conditions, (iii) the minimum type and quantity of equipment that can be used outside of the ICU, if needed, and (iv) whether the hospital prioritizes financial considerations above quality care.

The characteristics of having higher education and taking their patient to a private hospital were more frequent in the family members who expressed a lower level of satisfaction compared to those who expressed a high level of satisfaction. We learned that families with these characteristics are more demanding in relation to others. A higher level of dissatisfaction among family members with a college education emerged in other studies as well.^(3)^ The lower level of satisfaction with private hospitals can be attributed to the reasons mentioned above.

## CONCLUSION

This study found a low satisfaction index among the study participants from private and government hospitals. However, a relatively high satisfaction index score was noted in questions related to the level of care received by patients from the hospital staff, whether the intensive care unit staff members are courteous, and the satisfaction with the medical care that the patient received. A very low satisfaction index was observed for questions regarding potential improvements to the medical care that the patient received.

The results show a lower level of satisfaction for the majority of services offered by private hospitals. The use of a validated and reliable questionnaire in this study enhances the validity and generalizability of the findings to similar settings.

Further studies in this field must be encouraged to obtain a comprehensive assessment of the needs of the wider population and to establish the efficiency of tools designed to improve the level of satisfaction with the medical care and quality of care provided by intensive care units.

The prognosis of patients who were admitted to intensive care units and whether they survived and were released from intensive care units or died were not considered in this study. Hence, the effect of the prognosis as an independent factor was not assessed.

## References

[1] Chang JT, Hays RD, Shekelle PG, MacLean CH, Solomon DH, Reuben DB (2006). Patients' global ratings of their health care are not associated
with the technical quality of their care. Ann Intern Med.

[2] Jha AK, Orav EJ, Zheng J, Epstein AM (2008). Patients' perception of hospital care in the United
States. N Engl J Med.

[3] Roberti SM, Fitzpatrick JJ (2010). Assessing family satisfaction with care of critically ill
patients: a pilot study. Crit Care Nurse.

[4] Harvey MA (2004). Evidence-based approach to family care in the intensive care
unit: why can't we just be decent?. Crit Care Med.

[5] Heyland DK, Rocker GM, Dodek PM, Kutsogiannis DJ, Konopad E, Cook DJ (2002). Family satisfaction with care in the intensive care unit: results
of a multiple center study. Crit Care Med.

[6] Kosco M, Warren NA (2000). Critical care nurses' perceptions of family needs as
met. Crit Care Nurs Q.

[7] Neves FB, Dantas MP, Bitencourt AG, Vieira PS, Magalhães LT, Teles JM (2009). Analysis of family satisfaction in intensive care
unit. Rev Bras Ter Intensiva.

[8] Schleyer AM, Curtis JR (2013). Family satisfaction in the ICU: why should ICU clinicians
care?. Intensive Care Med.

[9] Alvarez GF, Kirby AS (2006). The perspective of families of the critically ill patient: their
needs. Curr Opin Crit Care.

[10] Wall RJ, Curtis JR, Cooke CR, Engelberg RA (2007). Family satisfaction in the ICU: differences between families of
survivors and nonsurvivors. Chest.

[11] Khalaila R (2013). Patients' family satisfaction with needs met at the medical
intensive care unit. J Adv Nurs.

[12] Johnson D, Wilson M, Cavanaugh B, Bryden C, Gudmundson D, Moodley O (1998). Measuring the ability to meet family needs in an intensive care
unit. Crit Care Med.

[13] Molter NC (1979). Needs of relatives of critically ill patients: a descriptive
study. Heart Lung.

[14] Fumis RR (2004). As famílias dos pacientes da UTI do Hospital do Câncer -
A.C. Camargo: suas necessidades e compreensão.

[15] Dodek PM, Wong H, Heyland DK, Cook DJ, Rocker GM, Kutsogiannis DJ, Dale C, Fowler R, Robinson S, Ayas NT, Canadian Researchers at the End of Life Network (2012). The relationship between organizational culture and family
satisfaction in critical care. Crit Care Med.

[16] Abvali A, Peyrovi H, Moradi-Moghaddam O, Gohari M (2015). Effect of support program on satisfaction of family members of
ICU patients. JCCNC.

[17] Heyland DK, Rocker GM, O'Callaghan CJ, Dodek PM, Cook DJ (2003). Dying in the ICU: perspectives of family members. Chest.

